# The Cognitive Aging of Episodic Memory: A View Based on the Event-Related Brain Potential

**DOI:** 10.3389/fnbeh.2013.00111

**Published:** 2013-08-26

**Authors:** David Friedman

**Affiliations:** ^1^Cognitive Electrophysiology Laboratory, Division of Cognitive Neuroscience, Columbia University Medical Center, New York State Psychiatric Institute, New York, NY, USA

**Keywords:** cognitive aging, episodic memory, familiarity, recollection, ERPs

## Abstract

A cardinal feature of older-adult cognition is a decline, relative to the young, in the encoding and retrieval of personally relevant events, i.e., episodic memory (EM). A consensus holds that familiarity, a relatively automatic feeling of knowing that can support recognition-memory judgments, is preserved with aging. By contrast, recollection, which requires the effortful, strategic recovery of contextual detail, declines as we age. Over the last decade, event-related brain potential (ERPs) have become increasingly important tools in the study of the aging of EM, because a few, well-researched EM effects have been associated with the cognitive processes thought to underlie successful EM performance. EM effects are operationalized by subtracting the ERPs elicited by correctly rejected, new items from those to correctly recognized, old items. Although highly controversial, the mid-frontal effect (a positive component between ∼300 and 500 ms, maximal at fronto-central scalp sites) is thought to reflect familiarity-based recognition. A positivity between ∼500 and 800 ms, maximal at left-parietal scalp, has been labeled the left-parietal EM effect. A wealth of evidence suggests that this brain activity reflects recollection-based retrieval. Here, I review the ERP evidence in support of the hypothesis that familiarity is maintained while recollection is compromised in older relative to young adults. I consider the possibility that the inconsistency in findings may be due to individual differences in performance, executive function, and quality of life indices, such as socio-economic status.

## Introduction

A great deal of experimental evidence indicates that older, relative to younger, adults exhibit a decline in episodic memory (EM) function, i.e., in the encoding and retrieval of personally relevant events (Light, 1991; Rugg and Morcom, 2005; Friedman et al., 2007; McDaniel et al., 2008). Over the last decade, the scalp-recorded event-related brain potential (ERP) has become an increasingly important tool in the study of the aging of EM for two reasons. First, ERPs have exquisite temporal resolution, in the millisecond range, and can, therefore, track the processing of mnemonic information at the speed with which those events transpire within the brain. Second, a few, well-researched ERP, EM effects have been associated with the cognitive processes thought to underlie successful recognition-memory performance (e.g., Yonelinas, 2002).

For example, Old/New recognition-memory tasks include a study phase followed by a delay, after which a recognition test is administered. Participants have to respond to a randomly intermixed series of previously studied (i.e., old) and unstudied (new) items, typically by quickly and accurately pressing a response button concordant with the old/new status of the item. At least two sets of processes are thought to contribute to performance on this type of recognition-memory task: familiarity and recollection. They have a long history of study in cognitive psychology (Mandler, 1980) as well as cognitive neuroscience (Yonelinas, 2002) and have played important roles in understanding age-related changes in EM (Jennings and Jacoby, 1993). Familiarity is thought to be fast acting and relatively automatic, with the majority of studies suggesting comparative preservation with aging (Howard et al., 2006). However, recent ERP (Duarte et al., 2006; Wang et al., 2012) and behavioral (Prull et al., 2006; but see Koen and Yonelinas, 2013 below) findings suggest that this might not always be the case. By contrast with familiarity, recollection takes longer to evolve, is deliberate and, therefore, thought to involve executive control. In behavioral studies, older, relative to young, adults consistently exhibit deficits in recollection-based processes (Jennings and Jacoby, 1993; Howard et al., 2006), possibly because they are impaired on tasks that tap executive-control functions (Braver and Barch, 2002; Buckner, 2004; but see Verhaeghen, 2011). In a very recent meta-analysis, Koen and Yonelinas (2013) came to the similar conclusion that, whereas recollection showed large decrements with aging, familiarity demonstrated small, though significant, reductions. In a follow-up experiment with participants between the ages of 40 and 81, these same investigators (Koen and Yonelinas, 2013) used several methods to estimate familiarity and recollection. Again, recollection-based processing showed large declines with aging, whereas familiarity-based processing was preserved, with each estimating procedure yielding the same pattern of findings. The fact that all methods employed produced the same result is strong evidence for the hypothesis that recollection shows clear and consistent declines with aging, while familiarity, if reduced at all, exhibits a much smaller diminution and is more often preserved with aging.

A good example of the two sets of mnemonic processes is demonstrated by the following scenario that we all have experienced at one time or another: you see a face in the crowd and have an immediate “aha” response that you know this person (familiarity-based judgment; i.e., a feeling of knowing), but cannot immediately bring to mind, for instance, the person’s name, in what type of venue you met the person and his or her occupation (recollection of some of the previous episode’s contextual details). The recovery of that kind of contextual information may take several hundred milliseconds or even longer. Such differential timing of familiarity- and recollection-based processes cannot be easily studied with fMRI techniques because the hemodynamic response is quite sluggish and cannot resolve processes occurring within milliseconds of stimulus presentation.

However, recognition-memory processes have been well studied with ERP methods (Johnson, 1995; Friedman and Johnson, 2000; Mecklinger, 2000; Paller, 2004; Rugg and Curran, 2007). Familiarity- and recollection-based EM effects are operationalized by subtracting the ERPs elicited by correctly rejected new items (CRs) from those to correctly recognized, old items (Hits). It is important to note that the *difference* between these two ERPs presumably reflects EM retrieval phenomena, and it is the difference between old and new ERPs that is the critical measure in most of these investigations. Although decidedly controversial, the mid-frontal EM effect (also known as the FN400; Curran, 2000) is a positive component between ∼300 and 500 ms, maximal at fronto-central scalp sites, and thought by some to reflect familiarity-based recognition (see below for a description of the controversy). A subsequent positivity between ∼500 and 800 ms, maximal at left-parietal scalp, has been labeled the left-parietal EM effect. A great deal of data accrued over the last 20 years and a rather strong consensus suggest that this brain activity reflects recollection-based retrieval (Rugg and Curran, 2007). A third, positive EM effect that generally occurs during and/or following the diminution of the left-parietal EM effect and endures for several hundred milliseconds, has been associated with the evaluation and monitoring of the products of a retrieval attempt. This activity is focused over right-frontal scalp, has been linked to executive function and the prefrontal cortex, but may not reflect mnemonic processes *per se* (Hayama et al., 2008). It has been labeled the right-frontal EM effect (Friedman and Johnson, 2000). Because of space limitations, this review will consider only the first two EM effects, those that have been the most frequent subjects of study (for a review of ERP activity related to executive-control processes at retrieval, see Mecklinger, 2010).

**Figure 1:**
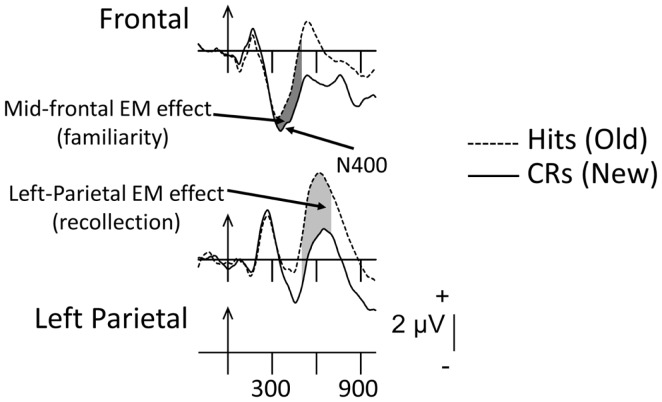
**Grand-mean ERPs averaged across 16 young adults to correctly recognized old items (Hits, dashed lines) and correctly rejected new items (CRs, solid lines)**. Arrows mark stimulus onset with vertical-hash, timing marks every 300 ms. Dark-gray shading in the waveforms indicates the mid-frontal effect (300–500 ms); light-gray shading reflects the left-parietal effect (500–800 ms).

Figure 1 depicts the ERPs associated with correctly recognized old items (Hits) and CRs in young adults and displays the two phenomena of interest. The mid-frontal and left-parietal EM effects are identified and the typical latency windows used to measure them are shaded (mid-frontal = dark gray; left-parietal = light gray). The reduction with repetition (i.e., an increment in positive amplitude) in the frontally oriented N400 can be clearly observed over frontal scalp. The functional significance of the difference between old and new ERPs in this region (usually measured between 300 and 500 ms) has been linked with familiarity but, as noted earlier, this interpretation is hotly debated. The subsequent enhancement in positivity (500–800 ms) to old compared to new items over left-parietal scalp can also be observed clearly. As noted, this EM effect has been associated with recollection.

The association between the mid-frontal EM effect and familiarity is based upon findings that its amplitude (1) is similar to hits regardless of whether they are endorsed with remember (R; recollection) or know (K; familiarity) judgments (Trott et al., 1999); (2) is similar to hits regardless of whether the contextual details from the previous experience are correctly identified (Friedman, 2004); (3) is similar to hits and falsely recognized items that are highly similar to previously studied old items, i.e., “lures” (Curran, 2000; Nessler et al., 2001); and (4) shows a graded relation with memory strength (i.e., level of familiarity; Woodruff et al., 2006; Wang et al., 2012). The longer-latency parietal EM effect has been associated with recollection because its amplitude (1) is larger to hits associated with R relative to K judgments (Smith, 1993; Trott et al., 1999); (2) is larger to hits associated with correct compared to incorrect source judgments (Wilding and Rugg, 1996); (3) is larger to hits compared to falsely recognized, but very similar lure items (Curran, 2000); and (4) is larger the greater the amount of information retrieved about the previous episode (Wilding, 2000; Vilberg and Rugg, 2009). Consistent with the mid-frontal and parietal EM effects reflecting distinct mnemonic processes, they differ in timing and distribution of amplitudes across the scalp (i.e., topography), suggesting that these effects are undergirded by at least partially non-overlapping neural networks (Johnson et al., 1998; Rugg and Yonelinas, 2003; Friedman, 2004).

In the review that follows, I will cover those age-related investigations that have been published since my last relatively comprehensive evaluation of the literature (Friedman, 2000). Although my colleagues and I included some review material in a later publication (Friedman et al., 2007), that paper was not a thorough assessment of the age-related memory and ERP findings. I will not include encoding-related ERP data because there have not been a sufficient number of papers in this area to come to a clear conclusion, although the handful of papers that do exist suggest a deficit in encoding-related processes (Nessler et al., 2006; Friedman, 2007; see also Johnson et al., 2013). I will also not discuss age-related studies of retrieval-cue processing (e.g., Morcom and Rugg, 2004), as these are outside the focus of this review. Similarly, continuous-recognition-memory (Walhovd et al., 2006), relative to Old/New paradigms, are known to depend upon distinctly different cognitive mechanisms (Friedman, 1990). Hence, these will also not be included.

I start with a brief description of the types of recognition-memory tasks that have been employed typically in studies of neurocognitive aging. Most ERP investigators of recognition-memory phenomena have used verbal items as to-be-remembered events. Hence, the possibility that the mid-frontal EM effect might reflect conceptual priming rather than familiarity cannot be ruled out definitively. Specifically, repeating a previously studied item during the recognition-memory test phase engenders a reduction of the N400 component (between ∼ 300 and 500 ms), which comprises one aspect of the mid-frontal EM effect (Figure 1). The N400 is associated strongly with semantic processing, i.e., it is conceptually based (Kutas and Hillyard, 1980). Moreover, some amnesic patients show deficits in familiarity-based processing during recognition-memory testing, while some of these same patients show intact conceptual priming (Olichney et al., 2000). Hence, because the vast majority of investigators of ERP memory-related phenomena have used words as memoranda, the processes involved in the conceptual priming shown by the amnesic patients and controls in the Olichney et al. (2000) experiment may overlap those reflected in the reduction of the N400 comprising one aspect of the mid-frontal EM effect. Therefore, rather than reflecting familiarity *per se*, the mid-frontal EM effect could be associated with conceptual priming. This is currently highly contentious (see Paller et al., 2012 and Mecklinger et al., 2012 for a thorough treatment of these competing positions).

All of the data reviewed below come from investigations of variants of the recognition-memory paradigm and are, therefore, thought to reflect EM processes. Hence, I will assume that, although familiarity and conceptual priming covary in most studies of recognition memory, the mid-frontal EM effect is a putative sign of familiarity-based processing. However, I will also attempt, in the summaries of each section, to determine whether conceptual priming could also have accounted for the results.

Additionally, some investigators (e.g., Nessler et al., 2007; Duverne et al., 2009), have interpreted their ERP data as indicative of “compensation,” in which older adults show electrical activity over different scalp regions compared to their young-adult counterparts (for fMRI data, see review by Grady, 2012). Compensation refers to the possibility of neural plasticity in healthy older adults, in which they may be able to reorganize neural networks (not recruited by the young) in order to cope with increased task complexity in the face of the deleterious effects of aging on the brain. Whether this hypothesis can be supported by the available ERP data is a topic that I will consider at the end of this review.

Three types of recognition-memory tasks have been used – simple, Old/New recognition, a more complex version of the Old/New paradigm, labeled the Remember (R)/Know (K) paradigm (Tulving, 1985), and the “source” memory task (for descriptions, see immediately below). Cued-recall paradigms have also been used (e.g., Angel et al., 2009), and I will discuss those studies in a separate section. In each of these paradigms, both pictures and words have been presented as memoranda. Surface format of stimulus material might be important in determining whether familiarity- as well as recollection-based EM effects are observed, especially in older adults, because pictures provide a much richer array of perceptual detail and enhance semantic elaboration to a greater extent than words (Yonelinas, 2002). Hence, I will add this distinction to the summaries of each section of the review.

During the test phase in Old/New recognition, one has to respond simply by judging whether the current item (i.e., the copy cue) is old or new, usually via reaction time (RT). To perform adequately on this task, either familiarity or recollection (or both) can be instrumental in reaching a decision. By contrast, in the R/K task, participants respond R if an old item is associated with any contextual details, be they inherent in the stimulus (e.g., semantic associates that are retrieved during encoding) or thoughts or ideas the person had during the time the item was encoded. A participant indicates K, when the item has been on the study list, but no contextual details can be recovered. R judgments are generally thought to reflect recollection-based decisions, whereas K judgments are thought to indicate familiarity-based decisions. As noted, in the R/K paradigm a wide variety of contextual details can underlie an R judgment. By contrast, in source-memory tasks, experimentally created, “diagnostic,” details or sources must be recovered during the test phase, although non-diagnostic details may also be retrieved. In these paradigms, recollection is thought to contribute more than familiarity, as it is believed that one must time travel back to the prior episode in order to recover the diagnostic, contextual information (this may also be true in the case of an R judgment). Both of these types of memory task might be considered “source” tasks, in which the nature of the sought-for information differs. Although this might seem obvious, mind-traveling back in time is most likely one of the reasons why recollection-based processes take longer to transpire than those involved in familiarity-based retrieval (McElree et al., 1999).

## Review of Studies

When available, I have indicated the age range of young- and older-adult samples for each of the studies I review below. When ranges weren’t available, I have inserted mean ages (±SD).

### Old/new recognition-memory paradigms

**Figure 2:**
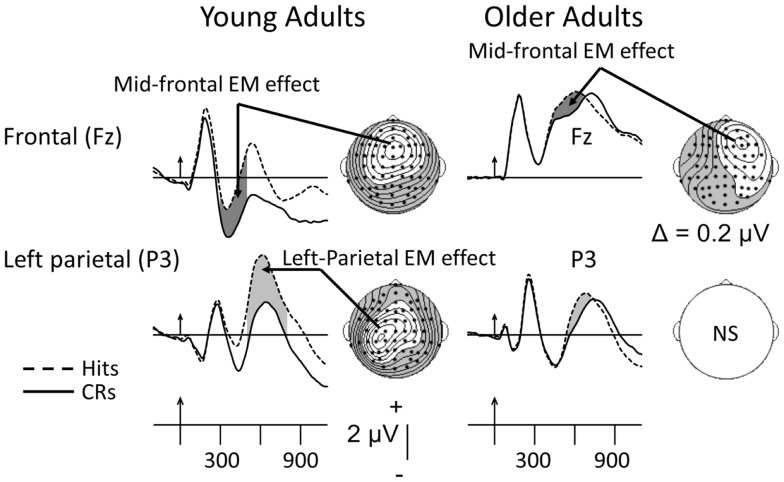
**Grand-mean ERPs averaged across 16 young and 16 older adults to correctly recognized old items (Hits, dashed lines) and correctly rejected new items (CRs, solid lines)**. Arrows mark stimulus onset with vertical-hash, timing marks every 300 ms. Dark-gray shading in the waveforms indicates the mid-frontal effect; light-gray shading reflects the left-parietal effect. NS, not significant. The maps here and in subsequent figures were computed by calculating contours using the spherical spline algorithm (Perrin et al., 1989) and data from all 62 electrodes (dots). In the topographic maps, unshaded regions reflect positivity; shaded regions reflect negativity. The data have been adapted from Nessler et al. (2007). The mid-frontal and left-parietal maps for the young were computed, respectively, between 300–500 and 500–800 ms. Due to latency prolongation in the older adults, the mid-frontal EM effect was computed between 400 and 600 ms.

As noted earlier, two old minus new EM effects have been the most heavily researched – the mid-frontal and left-parietal. These are depicted from an investigation by Nessler et al. (2007), whose data can serve to summarize the age-related findings for putative recollection-based neural activity recorded during the test phases of Old/New recognition-memory paradigms. This is so because similar age-related differences in the magnitude of recollection-related electrical activity have been reported by several other investigators (reviewed below). The data in Figure 2 were recorded from 16 young (18–29 years old) and 16 older adults (62–86) in a simple, Old/New recognition-memory paradigm. Participants viewed a list of words and then, following a 5-min delay, saw the same set of “old” words intermixed randomly with a set of new words. During the test phase, subjects were asked to judge whether the items were old or new via choice, speeded and accurate old/new button presses. Figure 2 shows that both young and older adults exhibit intact and significant mid-frontal EM effects, putatively reflecting familiarity. By contrast, Figure 2 shows that only the young adults display a reliable left-parietal EM effect, presumably reflecting recollection-based processes.

Wolk et al. (2009) also used a standard Old/New recognition task and found that young (18–30) relative to older (65–85), adults produced a greater magnitude recollection effect with words as stimuli. The mid-frontal effect was also reduced in the older-adult group. However, in similar fashion to Friedman et al., 2010; see Discussion below), when these investigators categorized their older adults into good and poor performers, only the old-high group showed neural evidence of recollection-based processing. Nonetheless, as in some of the other investigations described below, even the old-high, relative to the young-adult, recollection effect was still diminished in magnitude, suggesting that older adults recover less contextual information than their young-adult counterparts (Jacques St. and Levine, 2007).

Relative to words, pictorial stimuli are especially rich in perceptual detail and, hence, might be better recollected (i.e., the pictorial superiority effect) in both young and older adults. Nonetheless, although Gutchess et al. (2007) used full-color photographs of outdoor scenes as memoranda, their findings add to the evidence that older (61–74), relative to young (18–26), adults are impaired at recollection-based processing. Similar to the results of the Wolk et al. (2009) investigation, the mid-frontal EM effect was absent in the older-adult waveforms in the Gutchess et al. (2007) study. Ally et al. (2008b) compared directly, in young (18–25) and older (69–83) adults, picture–picture (study-test) with word–word recognition memory. Unlike the Gutchess et al. (2007) and Wolk et al. (2009) findings, the mid-frontal EM effect was of similar magnitude in young and older adults in the picture–picture condition, but was smaller in older, relative to young, adults in the word–word condition. Also in contrast to the results of Gutchess et al. (2007), Ally et al. (2008b) observed similar-magnitude recollection-based neural effects (and memory accuracies) in the picture–picture test condition in young and older adults, whereas in the word–word condition, young adults produced greater amplitudes (and memory accuracies) than older adults. Ally et al. (2008b) reported that there were no reliable topographic differences between young and older adults in the picture–picture condition. However, my visual impression was that older adults exhibited a strongly right-lateralized effect over frontal scalp (see also the section on Source-Memory and R/K Paradigms below), whereas the young adults showed the typical left-parietal scalp distribution associated with recollection.

In a follow-up investigation, Ally et al. (2008a) also employed pictures of common objects. During study, all objects were presented in canonical view. At test, all old items and an equal number of new items were presented for Old/New recognition testing. During the test phase, one-third of the old items were presented in the same canonical view as at study, one-third were rotated by 90°and one-third were presented in non-canonical views (see also Ranganath and Paller, 1999). Participants were to state “old” to studied items, regardless of the test object’s viewpoint. Ally et al. (2008a) focused on the recollection-related effect and did not assess the mid-frontal EM effect. Their young-adult (18–25), recollection-based effect magnitudes (500–800 ms) were ordered as follows: canonical > rotated > non-canonical. This finding adds to the evidence that the parietal EM effect reflects recollection, because the match in retrieved information between the canonical copy cue and the study item is greater than that between the non-canonical copy cue and the study object. In accord with the ERP data, overall memory accuracy was better in the canonical than the rotated and non-canonical conditions and, relative to older adults (62–83), young adults’ memory sensitivity was reliably better in all three conditions. Compared to the young, older adults demonstrated smaller parietal EM effects in all three conditions. However, older adults did not appear to produce significant recollection-based EM effects in any of the three conditions, consistent with the results of some of the studies reviewed earlier.

Like pictures, famous faces might also be expected to yield robust recollection-based processing to the extent that biographical features (i.e., the contextual details) are retrieved when the face is presented. Hence, Guillaume et al. (2009) used famous French faces as memoranda and assessed EM effects in young (25–30), middle-aged (50–64), and older adult (65–75) samples to determine when in the older age span declines in EM might begin. Although these authors collected R and K responses, they did not depict or analyze their data according to these judgments. The major findings were that (1) young, middle-, and older-aged participants showed similar memory accuracies; (2) the young and middle-aged groups both exhibited reliable mid-frontal EM effect effects, whereas the older adults did not; and (3) the young showed a reliable recollection-based EM effect, whereas the middle-aged sample’s was marginal and the older-adults’ effect was not significant. Unfortunately, there is some difficulty in assessing the validity of these findings because, for both middle-aged and older-adults, there was a high degree of measurement overlap between the mid-frontal and left-parietal temporal intervals.

To summarize, with a wide variety of memoranda, the Old/New recognition-memory data reviewed above suggest that putative recollection-based processing (as reflected by the left-parietal EM effect; Figure 2) is reduced as healthy individuals grow older, with that decline possibly beginning in middle-age. However, because of the overlap in time windows in the Guillaume et al. (2009) study, this latter result may be questionable. The evidence as to whether older adults evince preservation of familiarity-based processing is equivocal, as there is inconsistency in the presence or absence of the putative familiarity effect in the waveforms of older adults, at least in the Old/New paradigms reviewed above. Furthermore, although the richness of perceptual details inherent in pictorial objects relative to words might be expected to elicit larger-magnitude familiarity- and/or recollection-based activities, the findings in older adults are too variable to support this distinction. Moreover, the results also do not appear to provide evidence, in any straightforward manner, for the interpretation that the mid-frontal effect can be explained by the conceptual priming between study and test items. For example, the differential effect of aging on the surface format of study-test pairings (i.e., picture–picture vs. word–word in Ally et al., 2008b), would be difficult to reconcile with a conceptual priming account of mid-frontal activity (see also Wang et al., 2012, below). Additionally, a recent review of the age-related status of conceptual priming concluded that this function was unaffected by aging (Fleischman, 2007). Hence, if the mid-frontal EM effect’s magnitude were to change with aging, this would provide evidence against the view that this EM effect reflects conceptual priming. A further difficulty with more definitive interpretations of the EM effects recorded in canonical Old/New recognition paradigms is that behavioral proxies for familiarity and recollection have not typically been collected. This precludes the association of changes in magnitude and/or topography of a given EM effect with such proxy indices. This situation is remedied somewhat when source-memory and R/K investigations are considered.

### Source-memory and R/K paradigms

Generally, older adults perform more poorly, relative to tests of “item” or content memory, on tests that necessitate the recovery of contextually based information (for review, see Spencer and Raz, 1995). This age-related finding is typical of all of the studies reviewed below. That is, when considering the accuracy data collapsed across source correct and incorrect judgments (i.e., Total Hits), older, relative to young, adults are not as impaired compared to when only correct-source judgments are considered. In the canonical ERP source-memory experiment, participants first produce an old/new, “item” judgment in response to the copy cue. Then, for any item judged to be “old,” a short delay follows, after which an additional “source-memory” decision is given concerning which source (e.g., gender of voice; list membership; color of studied word/picture) the item was associated with during the encoding stage (e.g., Wegesin et al., 2002; Wilding and Rugg, 1996). It has been demonstrated that this “two-response” procedure generates highly similar ERP results as the one-response procedure, in which a Source 1, Source 2, or New judgment is made immediately following the presentation of the copy cue (Senkfor and Van Petten, 1998).

**Figure 3:**
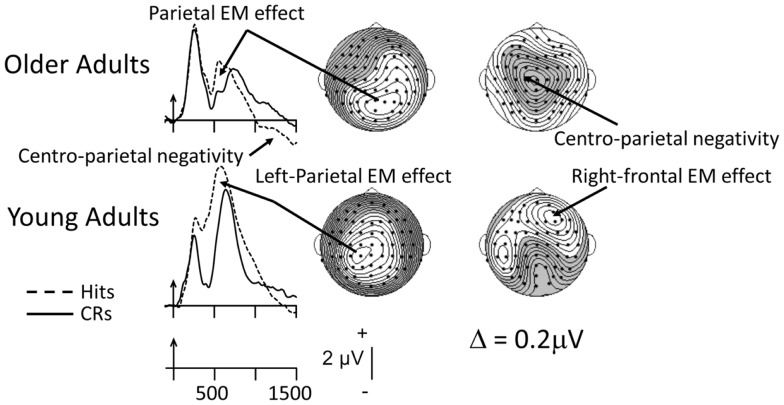
**Grand-mean ERPs averaged across 14 young and 14 older adults to correctly recognized old items (Hits; dashed lines) and correctly rejected new items (CRs, solid lines)**. Arrows mark stimulus onset, with time markers every 500 ms. The data were recorded during a source-memory paradigm in which two lists of sentences (i.e., the “sources,” each with two nouns) had been presented (Wegesin et al., 2002). Participants had to decide whether the nouns were new or old and, for old items, which list the noun had come from. The ERPs are depicted at the left-parietal site where the parietal EM effect has been consistently identified (Friedman and Johnson, 2000). The waveforms associated with correctly recognized old items (Hits) have been averaged across the two nouns from each sentence and each list (or source). The topographic maps illustrating the parietal EM effects for young and older adults were based on a latency window from 500 to 600 ms; the latency window for the subsequent effects was based on a 1000–1100 ms time interval. The data have been adapted fromWegesin et al. (2002).

Unlike the picture from Old/New recognition tasks, the age-related pattern of findings is somewhat different when source-memory data are considered. The ERPs depicted in Figure 3 provide an example of such data from a “two-response” procedure, in which participants first made an old/new judgment and then, for items judged old, generated a source decision (was the item presented in List 1 or List 2? see Wegesin et al., 2002, for complete details). The ERP data illustrated in Figure 3 were recorded time-locked to the copy cue. Young adults (18–28) show the typical left-sided, posterior scalp distribution associated with recollection-based activity. By contrast, the older adults (60–80) exhibit a posterior-parietal topography, but it appears to be somewhat right-sided compared to that of the young (see also Duverne et al., 2009). This relatively anomalous distribution might well be due to the overlapping centrally oriented negativity that is prominent in the ERPs of the older adults (for other examples see Li et al., 2004, and Swick et al., 2006, and Discussion below). In the same time frame (1000–1100 ms), in the young-adult ERPs there is posterior-negative activity, but the most conspicuous feature of their distribution is the right-prefrontal EM effect thought to reflect post-retrieval monitoring and evaluation. In the Wegesin et al. (2002) study, the difference between young and older adults in parietal EM effect magnitude was not significant. Correspondingly, young and older adults displayed significant, similar-magnitude mid-frontal effects (data not shown). On the other hand, older adults exhibited a central negativity which was not present in the ERPs of the young. Wegesin et al. (2002) speculated that the central negativity in older adults may have reflected the re-representation of the nouns’ visual images (Cycowicz et al., 2001), because several participants had used visualization strategies during encoding to memorize those stimuli. Wegesin et al. (2002) also suggested that this activity may have been “compensatory” because it was not present in the ERPs of the young. However, that argument does not rest on solid ground, as these authors did not attempt to relate the so-called compensatory activity to performance measures. I will come back to this point after reviewing other data that have come from similar source-memory paradigms.

Using a one-response test procedure, Li et al. (2004) had their young (18–24) and older (63–75) participants judge whether they had previously seen a picture and made a size judgment at encoding, whether they had previously viewed a picture and made a living/non-living judgment during study or whether the picture was new. Li et al. (2004) did not assess the mid-frontal EM effect. Rather, as this was a source-memory procedure in which presumably contextual detail had to be recovered in order to perform adequately, they concentrated on the ERP sign of recollection. In a condition in which performance was equated between young and older adults, the typical left-sided parietal EM effect was observed in young adults when comparing the ERPs to source-correct with those to CRs. By contrast, in older adults, there was no sign of the left-sided, recollection-based effect due to a large, overlapping left-sided negativity. However, in similar fashion to the Wegesin et al. (2002) data, over the right-hemiscalp, older, relative to young, adults showed equivalent magnitude parietal activity. Li et al. (2004) interpreted this to mean that, although the recollection-based effect was absent over left hemiscalp, the right-sided effect most likely reflected similar retrieval processes. As had also been suggested by Wegesin et al. (2002), Li et al. (2004) posited that the older-adult negativity reflected the reliance on and recovery of visual detail, based on data from investigations by Cycowicz et al. (2001), Friedman et al. (2005), and Johansson et al. (2002). In other words, they speculated that while young adults most likely used a conceptually based retrieval strategy, older adults recruited a fundamentally different, perceptually based strategy. Whether this could have reflected a “compensatory” brain response was not considered by these authors.

A more explicit interpretation of additional brain activity in older adults as compensatory was proposed by Swick et al. (2006). In their study, Swick et al. (2006) also recruited patients with frontal-lobe lesions with which to compare their older adults, as some authors have suggested that there is a qualitative similarity between these patients and older adults on certain aspects of EM performance (Stuss et al., 1996). Swick et al. (2006) used the two-response procedure described earlier. Unfortunately, these investigators did not measure their waveforms using the typical latency window (300–500 ms) when the effects of familiarity/conceptual priming are thought to occur (their window was 400–800 ms, more typical of the recollection effect). Nonetheless, relative to young adults (18–27), older adults (63–82) did not show evidence of the neural sign of recollection-based processing, not even over right-parietal scalp. Rather, in similar fashion to the data of Li et al. (2004) and Wegesin et al. (2002), a large, left-frontal negativity was present in the older-adult waveforms during the 400–800 ms interval, most likely reducing any left-sided, recollection-based effect that may have been present. The authors interpreted the presence of the negativity as reflecting “compensatory” brain activity which, if true, may have been ineffective, as the older adults performed reliably worse than their young-adult counterparts.

Interindividual variability in performance and ERP measures in older adults may indicate that EM decline is not an inevitable aspect of cognitive aging. Data that support this notion from an R/K-source-memory paradigm have been reported by Duarte et al. (2006). Young (18–25) and older (60–83) participants made R/K/New decisions and then, for any item judged R, indicated whether the picture had been studied under manipulability or animacy encoding instructions. Duarte et al. (2006) categorized their older-adult participants on the basis of their memory-sensitivity performance into old-high (equivalent performance to young adults) and old-low (lower performance than young adults) subgroups. Contrary to most studies of cognitive aging, older adults as a whole showed lower familiarity estimates than young adults (but, see discussion of Wang et al., 2012, below), whereas estimates of recollection were similar in old-high and young adults but, as one might expect, lower in old-low adults. The mid-frontal and recollection-based EM-effect data were fairly consistent with the behavioral data – young adults produced reliable signs of these processes, whereas both old-high and old-low subgroups did not show significant mid-frontal EM effects. By contrast, old-high participants showed a reliable recollection-based EM effect with a scalp distribution and magnitude similar to that of young adults. However, although old-low subjects displayed a robust old/new effect between 700 and 1200 ms, this difference was negative-going over left-frontal scalp, and was not present in the ERPs of the young or old-high adults. Duarte et al. (2006) considered whether their negative-going effect might have reflected compensatory brain activity to counter the reduction in both familiarity- and recollection-based processing, or “dedifferentiation,” in which the old-low subgroup, unlike the young and old-high adults, could have recruited neural networks not specialized for the task at hand (see Reuter-Lorenz and Park, 2010, for further details). Duarte et al. (2006) could not come to a firm conclusion concerning which of these alternatives was more likely. Furthermore, as was true of the data of Wegesin et al. (2002), these investigators did not attempt to correlate the magnitude of this activity with mnemonic performance.

A similar decrement, relative to young adults (20–25) in the older-adult (61–81) recollection effect was observed by Friedman et al. (2010) in a source-memory paradigm in which initially meaningless, symbol-like objects were presented for study. By contrast, the mid-frontal EM effect appeared to be intact in these older adults. However, when Friedman et al. (2010) categorized their older adults into good and poor performers on the basis of memory sensitivity, only the old-high subgroup showed evidence of the mid-frontal and left-parietal EM effects; they were not present in the ERPs of the old-low subgroup. Rather, the ERPs of the low-performing older group were characterized, as in the Duarte et al. (2006) investigation, by a left-frontal negative EM effect (∼600–900 ms) that could have reflected compensation for the reduced familiarity- as well as recollection-based processing in this group. As the data were preliminary and the two subgroups had small *N*s (*N* = 8), these data need to be considered with caution.

The majority of the investigations of source-memory reviewed above employed sources that were most likely not chosen on the basis of older adults’ performance and, therefore, might not have been optimal for inducing good contextual-memory retrieval in these participants (all sources are not created equal; see Spencer and Raz, 1995). In an attempt to boost the source-memory performance of their older adults (59–75) relative to young adults (19–29), Dulas et al. (2011) used contexts manipulated by the type of judgment made during encoding while participants viewed pictures of common objects. They hypothesized that the self-referential nature of pleasantness judgments (is this item pleasant to you?) relative to self-external, “commonness” judgments would enhance the source-memory performance of older adults (Symons and Johnson, 1997). Although pleasantness relative to commonness judgments led to significantly greater source accuracy in both older and young adults, young adults still reliably outperformed their older-adult counterparts during the retrieval phase for words encoded under both conditions. Intriguingly, however, the neural sign of recollection was of equivalent magnitude in young and older adults, although the mid-frontal EM effect was smaller in older adults. Importantly, recollection-based neural activity was larger in the self-referential compared to the self-external condition, but only in older adults. Hence, it appears that, at least from the ERP data, older adults benefited more from the encoding manipulation than did young adults. Based on the interpretation that the recollection-related effect reflects the amount of contextual detail recovered from EM (Wilding, 2000; Vilberg et al., 2006), this finding suggests, by contrast with most age-related investigations, that the number of details recollected was greater in older adults for correct-source judgments associated with self-referential compared to self-external experiences.

In a similar attempt to employ episodes that were likely to be as well remembered in older (*M* = 68.3; SD = 2.7) as in young adults (*M* = 22.7; SD = 2.5), Eppinger et al. (2010) first asked participants to learn common-object-reward pairings (positive, +50 cents; negative, −50 cents; neutral, 0). Volunteers were given 15 trials of each object-reward pairing and then were administered an Old/New recognition series for the objects. For any item judged “old,” they then had to state the “source,” i.e., was the object associated with positive- or negative-feedback. Their experiment was based on the finding that older adults tend to remember stimuli that have positive valences, relative to those that are negative. Eppinger et al. (2010) suggested that this implied that older adults have a positive memory bias for self-relevant information. Unlike many previous investigations, older adults showed equivalent memory sensitivity during Old/New recognition as well as source-memory, most likely as a result of the large number of repetitions during study. The mid-frontal EM effect (250–400 ms) was of similar magnitude in young and older adults, but only for objects that had been paired with positive feedback. By contrast with previous studies, the putative recollection-based effect (450–700 ms) was characterized by a right-frontal topography in young as well as older adults for correct-source judgments to objects associated with both positive- and negative-feedback. However, consistent with several of the studies reviewed above, while the young adults also exhibited an EM effect at left-parietal sites for both types of feedback (between 450 and 700 ms), this left-sided activity was absent in the ERPs of the older adults. The right-frontal nature of the scalp distribution during the 450–700 ms interval renders unclear its association with recollection (but, see the interpretation by Li et al., 2004), although the concomitant left-sided positive activity does imply such a relation, at least in young adults. Perhaps, as Eppinger et al. (2010) posit, positive feedback has a greater effect on familiarity- than recollection-based recognition. On this view, older adults might have been able to base their judgments on familiarity rather than recollection, presumably accounting for their, respectively, intact and absent, mid-frontal and left-parietal EM effects. The authors concluded that the findings suggested that their older adults attributed a greater degree of emotional valence to positive feedback (the “positivity effect,” see Mather and Carstensen, 2005) during memory acquisition, which may have counteracted older adults’ well-documented decline in source memory.

To obtain a better handle on age-related changes in the putative familiarity-related EM effect, Wang et al. (2012) employed a modified R/K procedure with words based upon the hypothesis that familiarity relies on a graded memory-strength signal (Yonelinas, 2002). In the procedure used by Wang et al. (2012), R judgments are assumed to reflect recollection-based retrievals. Rather than using a single “Know” judgment (which could include retrieval of items with widely varying memory strengths), Wang et al. (2012) sorted “non-recollected” old items (i.e., those not given an R judgment) according to the confidence rating participants assigned to these items – confident old, unconfident old, unconfident new, and confident new. Based on these data, unlike the majority of behavioral data noted earlier, both familiarity- and recollection-based behavioral estimates were lower, relative to young adults (18–28) in older adults (63–76). While their young adults showed a gradation in mid-frontal EM effect magnitude (300–500 ms; R > confident old > confident new), these conditions did not differ in the ERPs of the older adults, nor did older participants produce a reliable mid-frontal effect (as was also the case in Duarte et al., 2006). By contrast, for both young and older adults, the recollection-based EM effect (500–800 ms) was significantly larger to items given an R judgment relative to confident old and confident new judgments (the latter two did not differ for either group). Of note, and consistent with several previous reports, the older, relative to the young, adults produced a reliably smaller recollection-based effect. The major conclusion reached by Wang et al. (2012) was that, relative to young adults, the putative familiarity-based retrievals of older adults may have depended on qualitatively different cognitive mechanisms that might not have been observable at the scalp.

In sum, the results of source-memory investigations suggest a more complex picture than that emerging from studies of recognition memory. Nonetheless, when it has been measured, the mid-frontal EM effect findings again indicate a great degree of between-study variability. This effect’s magnitude can be equivalent in young and older adults, or smaller in older adults, as well as absent in older relative to young adults, the latter occurring even in high-performing subgroups of elderly individuals (e.g., Duarte et al., 2006). This variability mirrors that observed in many investigations of cognitive aging, especially when age groups are categorized on a variable that declines with age (e.g., executive function; working-memory capacity). I will consider age-related variability in a separate section of the discussion below. Like the recognition-related data reviewed earlier, the source-memory findings do not appear to support, in any simple fashion, a role for conceptual priming in modulating the magnitude of the mid-frontal effect. For example, it would be difficult to reconcile the conceptual priming account with the Wang et al. (2012) finding of a relation between graded-confidence and mid-frontal effect magnitude. Finally, the data are also equivocal with respect to whether the retrieval-related brain activity of older adults benefits more from pictorial than verbal stimuli as memoranda.

The results for the putative recollection-based EM effect are somewhat more consistent. Several source-memory studies have revealed the presence of either a centrally- or left-frontally focused negativity (larger to old than new items) that tends to overlap and thereby reduce the magnitude of any left-parietal EM effect that might be present. This negative-going activity is thought by some investigators to be compensatory (but see Discussion below). However, in these situations, older adults tend to produce right-parietal activity that has been interpreted as reflecting the same retrieval operations as its left-parietal counterpart (Li et al., 2004).

### Stem cued-recall

Free-recall is arguably the ultimate and most valid technique for assessing recollection-based processing, as neither a copy cue nor a word-stem is available to guide retrieval. Hence, the participant must engage a conscious, effortful strategy for mind-traveling that will maximize the number of items recalled. Generally, older, relative to young, adults perform worse on free-recall than they do on recognition (Craik and McDowd, 1987). Word-stem cued-recall is a step removed, as the three-letter stem serves as a cue to aid retrieval of the complete word (that is, the word that was on the study list). Although previous authors have investigated the brain’s electrical activity in young adults in this type of paradigm (e.g., Allan et al., 1996, 2000), to my knowledge, only Angel and colleagues have used the word-stem, cued-recall task in age-related studies (Angel et al., 2009, 2010, 2011). In the Allan and colleagues’ studies with young adults, a positive-going, cued-recall EM effect was observed that exhibited an early left-sided, temporo-parietal scalp topography, and a subsequent right-frontal scalp distribution. Nonetheless, the cued-recall effect behaved similarly to the left-parietal recollection effect elicited in standard Old/New and source-memory recognition tasks. That is, it was larger to items studied under deep than shallow semantic-encoding conditions (Rugg et al., 1998; Allan et al., 2000), and to correct compared to incorrect source retrievals (Allan and Rugg, 1998). Allan et al. (2000) concluded that the cued-recall EM effect received contributions from the generators giving rise to the left-predominant, recollection effect as well as those responsible for producing the longer-duration, right-prefrontal EM effect. Because cued-recall necessitates the reinstatement of a word given only the word’s stem, it arguably recruits greater executive control processes relative to recognition. This might account for the presence of the right-frontal EM effect.

An important methodological feature of the word-stem, cued-recall paradigm is the ability to distinguish between an “explicit” (i.e., episodic) and an “implicit” memory retrieval (i.e., due to repetition priming). The former is operationalized as completing a word-stem with an item that was on the study list and is judged to be “old.” The latter is defined as completing a word-stem with an item that was on the study list but is unrecognized as having been on that list (Allan et al., 1996). Another important feature in these studies is the “baseline” completion condition, i.e., word stems completed with an appropriate word, but one that was not on the study list and is judged correctly to be “new.” One shortcoming in all of the Angel et al. studies reviewed below is that these investigators did not compute putative “implicit” word-stem completion averages. Hence, they could not comment on the presence in the electrical record of components that might have reflected such, presumably, non-conscious, implicit retrievals. In the first investigation by Angel et al. (2009) using the stem-cued paradigm, these authors compared the ERPs elicited by correctly rejected, baseline stem completions with those stems completed by studied items correctly recognized as old. Angel et al. (2009) observed putative recollection-based EM effects in the young- (*M* = 21; SD = 1.9) as well as older-adult (*M* = 65; SD = 3.3) age group, which did not differ in magnitude. However, no scalp maps were available to determine the similarity of these effects to those already published. Nonetheless, Angel et al. (2009) claimed that, whereas the young adults’ topography was left-sided, that of the older adults was bilaterally distributed. They noted that this asymmetry was similar to the HAROLD pattern (Hemispheric Asymmetry Reduction in the Old) observed in some of the hemodynamic data of older adults (Cabeza, 2002) and, on this basis, suggested that older adults had “compensated.” Nevertheless, it remains unclear what the older adults had compensated for, or whether such “compensation” was effective, because these participants performed reliably more poorly than their young-adult counterparts. Further, no attempt was made to relate this activity to performance measures.

In an attempt to obtain more information on variability in the older-adult population, in a second investigation using the same paradigm, Angel et al. (2010) explored the relations between educational level (often used as a proxy for cognitive-reserve; Stern, 2002) and behavioral word-stem, cued-recall performance, and ERP EM effects. Angel et al. (2010) divided their young (*M* = 25; SD = 1.9) and older (*M* = 66; SD = 5) participants at the median into low- (LE) and high-education (HE) groups, each with *N*s of 14. The major finding was that the older-adult HE group showed better memory accuracy than its LE counterpart. The effect of education was not significant for the young, most likely due to the greater homogeneity in this group. The major ERP finding was that, relative to the LE group, both young- and older-adult HE groups showed larger putative, recollection-based parietal EM effects. Nonetheless, the young HE recollection effect was reliably larger than that of the old HE group suggesting, as noted earlier that, relative to the young, even high-performing older-adult groups may recover fewer contextual details during retrieval. These data add to the currently limited ERP evidence that older-adult samples cannot be considered homogeneous, and that level of education may exert a positive effect on the memory performance and associated brain activity of some older-adult individuals (see also, Czernochowski et al., 2008).

In a second exploration of age-related variability in neurocognitive indices, Angel et al. (2011) again employed the word-stem, cued-recall paradigm. As in their previous study (Angel et al., 2009), Angel et al. (2011) observed left-lateralized recollection-based EM effects in their young participants (23–26) but bilaterally symmetrical effects in their older adults (60–80), which they again interpreted as compensatory within the framework of the HAROLD model (Cabeza, 2002). Relative to older adults, the left-sided, young-adult recollection-based effect was significantly larger. However, as best as can be determined, the presumed recollection-based EM effect was only reliable for the young adults and was not significant for the older adults, in accord with other studies reviewed in this section.

In summary, although only explored by a single laboratory, the word-stem cued-recall data suggest that, relative to young adults, older adults retrieve a smaller amount of information when correctly completing a stem with a previously studied word. Hence, these data join those resulting from recognition-, source-, and R/K-memory experiments. On the other hand, it would be helpful to have other, independent, laboratories confirm these age-related, word-stem, cued-recall findings. Nonetheless, whether the right-lateralized activity observed in older participants in these cued-recall investigations is truly compensatory has not been vigorously tested (see section on Compensation below).

## Discussion

### Familiarity vs. recollection

If the presumption that the left-parietal EM effect reflects the amount of contextual detail recovered from EM is valid, then the majority of studies reviewed above suggest, as do their behavioral counterparts, that recollection-based processing is deficient in older adults. In several studies, this magnitude reduction has been associated with lower performance in older-adult samples. Nonetheless, even when performance was matched (e.g., Li et al., 2004), or high-performing older adults were compared to young adults (Angel et al., 2010), these older individuals still exhibited smaller recollection-based EM effects (but see Duarte et al., 2006). Then again, the picture of the cognitive aging of familiarity-based processing is less clear. This state of affairs is due, in no small measure, to the current controversy concerning whether the mid-frontal EM effect reflects familiarity and/or conceptual priming. Hence, even if the magnitude findings were consistent (which they clearly are not) it would be difficult to come to a definitive conclusion. Blurring the picture even further is the fact that the behavioral findings point clearly to the preservation or minimal disruption of both episodic familiarity-based processing (e.g., Howard et al., 2006) and conceptually based implicit memory (i.e., priming; Monti et al., 1996; Fleischman and Gabrieli, 1998; Fleischman, 2007). This makes the absence of the mid-frontal EM effect in some studies difficult to understand (but, see Wang et al., 2012 for one interpretation). Adding to the problem of reaching an informed conclusion is the fact that, with few exceptions (e.g., Wang et al., 2012), many investigators have assumed that the mid-frontal EM effect reflects familiarity without collecting behavioral proxies that could validate the presence of this type of processing (e.g., R/K judgments). To disambiguate these two potential contributors to the processes reflected by the mid-frontal EM effect might also require a conceptual-priming manipulation, as has been argued for by Paller and his associates (e.g., Paller et al., 2012). This is especially true of the studies of the canonical Old/New recognition-memory paradigm, in which most investigators have used the mid-frontal EM effect as a proxy for familiarity, without collecting a relevant behavioral measure that would enable them to conclude, on a more definitive basis, that this indeed was the case. Nonetheless, as noted earlier, it is difficult to reconcile the finding of a strong relation between confidence ratings and mid-frontal EM effect magnitude (e.g., Wang et al., 2012) with a conceptual-fluency account of the data (see also, Rosburg et al., 2011 and Mecklinger et al., 2012).

One possibility for the absence of a putative, familiarity-based neural signature in some of the studies reviewed above is that other, earlier-occurring processes contribute to recognition-memory decisions. For example, Tsivilis et al. (2001) reported that the amplitude of an early EM effect (between 100 and 300 ms), with a fronto-polar scalp distribution was more consistent with a familiarity-based effect than the mid-frontal EM effect, which was also present in their waveforms (see also, Duarte et al., 2004). This early latency effect is consistent with primate data (Brown and Bashir, 2002) that suggests that a “familiarity-based” signal can occur quite early, at around 100 ms, well before the peak of the human mid-frontal activity. Hence, it is possible that, in some of the age-related investigations reviewed above, investigators, choosing to measure the purported 300–500 ms “familiarity” interval, may have missed early onset, hit vs. correct-rejection differences. This bears future investigation.

In addition to familiarity and recollection, other mechanisms are known to contribute to the retrieval of information during recognition memory. For example, a perceptual, implicit-memory mechanism might be responsible for the brain’s relatively automatic retrieval of a previously experienced event in the absence of conscious awareness about that episode. Though not without its difficulties, repetition priming is one way of operationalizing this putatively implicit or indirect influence. Because the processing fluency of an item is increased via repetition, increments in fluency can lead participants to judge an item as having been previously studied (Jacoby and Dallas, 1981). As older adults are relatively unimpaired on repetition-priming tasks relative to direct or explicit (i.e., episodic) memory (Friedman et al., 1993), one might expect the neural correlates of such processes to be preserved in older relative to young adults (to the extent that they can be observed at the scalp). Although some work in this domain has been performed with young adults (Friedman, 2004; Woollams et al., 2008; Yu and Rugg, 2010; Lucas et al., 2012), such data are missing in studies of cognitive aging. This could be a productive area of future research.

Two major, but alternative hypotheses have been advanced to explain the functional significance of the left-parietal EM effect elicited during the retrieval phases of recognition-memory paradigms: (1) it could reflect internal attentional orienting to mental representations retrieved from EM (see Vilberg and Rugg, 2008 for review); or (2) it could reflect neural activity that aids the online representation of recollected information (Vilberg and Rugg, 2009; Rugg and Vilberg, 2013), including the possibility that it might indicate the engagement of the episodic buffer postulated by Baddeley (2000) in his updated account of working memory (Vilberg and Rugg, 2008). Given the age-related data reviewed above, it would be difficult to argue for one interpretation over the other. However, the majority of the evidence, which is based solely on young-adult data, appears to support the second alternative. Nonetheless, the data on the viability of the episodic-buffer hypothesis is scarce. Hence, to the extent that the recollection-based EM effect indexes similar processes in young and older adults would imply that the older-adult recollection effect most likely indexes the amount of information (i.e., contextual details) retrieved from long-term memory. On this view, as noted previously, older adults do not appear to retrieve as many details as their young-adult counterparts.

### Compensation

The question of whether older adults recruit electrical activity that reflects “compensatory” processes to counteract deficits in mnemonic cognition was raised earlier. This idea, that older adults might bring “new” neural networks online (not recruited by the young) to thwart cognitive decline, was first observed in the PET/fMRI literature (e.g., Cabeza et al., 2002). This very attractive hypothesis implies plasticity in the aging brain, an idea that was, until the advent of neuroimaging, thought to be relatively untenable. However, while some authors argue that compensatory fMRI and ERP brain activity should only be evident in high-performing older adults (Cabeza et al., 2002; Riis et al., 2008), there are fMRI/PET and ERP data that show additional activity in *poorly performing* older adults (Fabiani et al., 1998; Nielson et al., 2002; Colcombe et al., 2005; Friedman et al., 2010). Hence, the issue of the functional significance of this type of additional brain activity is quite unsettled. Moreover, in many of these reports, precisely which cognitive processes are being compensated for is often not discussed. It is also unclear, in some investigations, whether the compensatory activity is correlated positively with performance, which arguably it ought to be, if such activity presumably benefits older-adult cognition.

These same criticisms apply to the limited ERP compensation-related data mentioned earlier. Nonetheless, ERP data may be better able to identify the kinds of processes reflected by such additional brain activity than slower techniques such as fMRI. For instance, if the compensatory activity is recruited to counter the reduction in recollection-based processing and enhance the recovery of information encoded in the previous episode’s memory trace, then that activity should most likely occur prior to the recognition-memory decision (Johnson et al., 2013). Indeed, in the Nessler et al. (2007) study described in the recognition-memory section, the retrieval-related, putatively compensatory, left-frontal negative-going activity preceded participants’ EM judgments by several hundred milliseconds. In fact, following up the Nessler et al. (2007) investigation, Johnson et al. (2013) elicited highly similar, retrieval-related, compensatory activity in young adults by disrupting episodic encoding (i.e., semantic elaboration) during the study phase. Like the Nessler et al. (2007) data, this retrieval-related activity had a scalp focus over the left inferior prefrontal cortex (LIPFC), a brain region implicated heavily in the control and retrieval of semantic information (e.g., Badre and Wagner, 2007). Similarly, the activity preceded the memory judgment by several hundred milliseconds and, importantly, its magnitude was correlated positively with memory accuracy. Hence, “compensation” is not limited to older adults. Rather, such activity can occur at any point in the lifespan, as suggested recently by Reuter-Lorenz and Park (2010) in their “scaffolding” account of compensation-related brain activity.

An inkling of the processes this activity might have reflected in the Johnson et al. (2013) investigation comes from an event-related fMRI study by Raposo et al. (2009), in which episodic retrieval was made difficult by limiting the amount of semantic information at encoding that could be integrated into EM traces. Similar to Johnson et al. (2013), though with better spatial resolution, these investigators observed compensatory activity over LIPFC at retrieval (i.e., this activity was correlated positively with performance) for items that were difficult to recover by virtue of the reduced semantic elaboration they had received during encoding. Raposo et al. (2009) interpreted this area of activation as indexing the recovery of episodic information that proceeded by highlighting the semantic memories that were generated during encoding. Because of the similarity in the topographic maps (Johnson et al., 2013) and areas of hemodynamic activation (Raposo et al., 2009) in the two investigations, Johnson et al. (2013) invoked a similar explanation to account for the compensatory activity that they observed over LIPFC.

Part of the difficulty in specifying which particular processes are invoked is due to the large disparities in cognitive ability between young and older adults and the use of young-elderly group comparisons to define and/or assess compensatory activity. Hence, our use of within-group comparisons of young adults with presumably intact cognitive abilities appears to have aided in elucidating the timing and nature of the underlying processes that might account for the presence of compensatory activity in young and older adults (see Johnson et al., 2013, for a complete discussion).

### Age-related variability

Finally, I come to a brief discussion of the greater variability typically associated with the performance and ERP data of older-adult samples (Morse, 1993). Several investigations of age-related change have noted increases in interindividual variability in older age groups (e.g., Frias et al., 2007). As we have seen, one investigative team (Duarte et al., 2006) used recognition-memory test performance to divide their older adults into low- and high-performing subgroups, and found large differences in recollection-based processing (favoring the high-performing subgroup) between the two older-adult subgroups. Similarly, Angel et al. (2010), following the cognitive-reserve hypothesis (see Stern, 2002 and discussion below), categorized their groups into those with low- and high-educational status and showed reliable effects of high-educational status in older adults on both performance measures and recollection-based brain activity (see Czernochowski et al., 2008 for a similar effect of socio-economic status in a recency/recognition paradigm). These data suggest that the cognitive-reserve hypothesis might provide a reasonable account for the increased variability observed in old age, although other hypotheses reviewed briefly at the end of this section might also explain these data.

**Figure 4:**
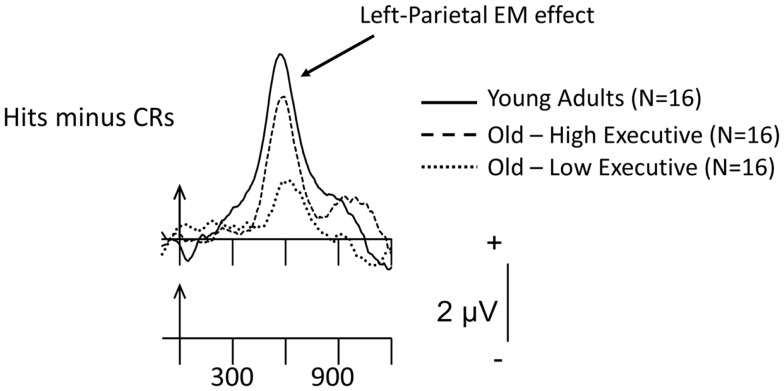
**Grand-mean Hits minus CR difference ERPs for items presented three times during study, averaged across the 16 young adults, 16 old-high executive function (EF), and 16 old-low EF subgroups at the left-parietal scalp site, P3**. Arrows mark stimulus onset, with time markers every 300 ms. The waveforms for the three groups have been superimposed. These preliminary data were recorded during the final recognition-memory test phase, which took place 1 h following the initial test series.

Our group has also taken a foray into this area of research in an attempt to determine if recollection-based processing would differ in older-adult samples categorized according to their executive-function performance. Because there are data indicating that some memory paradigms (for example, free-recall) require good executive skills to perform adequately (e.g., Taconnat et al., 2007) and, as noted earlier, older adults perform worse on free-recall compared to recognition (Craik and McDowd, 1987), our older-adult participants were categorized into those who were low- and high- on the basis of a series of executive-function assessments [the manipulation and maintenance of information in WM – assessed by reading and computation spans; task-set switching, a quintessential executive task; and the Eriksen flanker test (Eriksen and Eriksen, 1974), which yields a measure of inhibition]. Figure 4 depicts preliminary ERP data elicited during a study/test recognition-memory paradigm in which items were presented either one or three times during encoding and tested in initial (30-min following study) and final (1-h following study) recognition-memory assessments (Radin et al., unpublished observations). Note that the categorization of the older-adult data into low- and high-performers neatly orders the magnitude of the recollection-based parietal EM effect: Young > Old-High > Old-Low. This ordering also held for the memory sensitivity or accuracy of the three groups. Clearly, those older adults who scored well on the tests of executive function are those who produce the largest recollection-based electrical activity. Nonetheless, as has been noted throughout this review, the high-performing older adults do not reach the level of putative recollection-based processing shown by their young-adult counterparts. This again suggests that older, relative to young, adults do not recover the same amount of information when interrogating their EM traces. Although speculative, the larger recollection effect in the old-high, relative to the old-low, participants might be due to more efficient retrieval strategies, presumably instantiated in the prefrontal cortex and its interconnections where the computations involved in executive processes are thought to take place. On the other hand, the old-high group may have encoded the items more deeply, creating relatively richly detailed memory traces (again, with greater strategic control than their old-low counterparts), thereby rendering their recollection-based retrievals more facile. The similar or differential contributions of encoding and retrieval to age-related memory performance and brain activity are clearly questions for further, individual-difference ERP research.

A few theories have been advanced to account for the greater variability in older compared to younger adults. One of the most popular, the tenets of which were described earlier, is the compensation account (Cabeza et al., 2002), which has recently been modified and updated by Park and Reuter-Lorenz (2009) in their “scaffolding” model of compensatory brain activity. This latter theoretical stance posits that the recruitment of additional brain activity and, presumably, cognitive processes, is an adaptive response that can occur at any point along the lifespan (see Park and Reuter-Lorenz, 2009 and Reuter-Lorenz and Park, 2010, for reviews). A second, influential explanation is the cognitive-reserve hypothesis (Stern, 2002), which posits that certain factors (i.e., IQ, occupational, and educational status) provide buffers that mitigate the effects of age-related brain insult on cognitive function. This hypothesis suggests that older adults with high levels of reserve capacity (defined by these proxy measures) may be better able to maintain normal cognitive function throughout old age than their low-reserve counterparts (see, for an example, the Angel et al., 2010 investigation reviewed earlier). However, the division of the data into low and high groups based on some salient variable is silent about how long these differences have existed. For example, they may have been present since birth (possibly genetic) or since early childhood and young adulthood and maintained throughout middle and old age. The third hypothesis, one that is complimentary to that of cognitive-reserve, takes this possibility into account. The “brain maintenance” model of memory aging (Nyberg et al., 2012) postulates that those older individuals who have, since their young-adults days, maintained their neurocognitive abilities at high levels, are those who age “successfully.” On the other hand, at the current stage of knowledge it is entirely unclear which of these models can best account for the ERP/cognitive-aging data that have so far been collected.

## Conclusion and Future Directions

This survey of the ERP/memory and aging literature indicates that the neural evidence for disruption or preservation of familiarity (and/or conceptual priming) is extremely mixed and I can draw no firm conclusion at this time. On the other hand, the data suggest that, in many circumstances, recollection-based processing is diminished in older adults, in accord with much of the behavioral literature. However, there are clearly individual differences among older adults in the extent to which this facility is disrupted. The study of interindividual variability has a long history in cognitive-aging research (Botwinick and Thompson, 1968). However, from the perspective of the ERP technique, the unraveling of the underlying sources of age-related variability is clearly in its infancy. As a whole, the scant individual-difference data reviewed above, including putative “compensatory” brain activity, indicate, as highlighted by others (Duarte et al., 2006), that older adults cannot be considered a homogeneous population and that deficits in EM are not an inexorable consequence of aging. Hence, mnemonic function may be amenable to improvement through cognitive-training regimens (Lustig et al., 2009; Greenwood and Parasuraman, 2010). For example, one documented deficit is that some older adults do not engage in self-initiated processing to encode items into and retrieve items from EM because environmental support may be lacking (Craik, 2008). Hence, this might be one processing strategy that could be trained in older adults with documented memory deficits.

Additional investigations of “compensation” are needed to validate the construct (at least in the ERP domain), understand the nature of the antecedent conditions that lead older (and younger) adults to recruit novel neural networks and to understand the cognition that this extra brain activity reflects. Similarly, preliminary evidence from this laboratory (Czernochowski et al., 2008; Radin et al., unpublished observations; Figure 4) and others (Angel et al., 2010) indicates that some life-style variables, such as socio-economic status and level of educational attainment, as well as level of executive function may modulate the extent to which mnemonic processes are disrupted in older adults. Further investigation of the influence of these and similar variables should be undertaken. Similarly, the cognitive-reserve, compensation and brain-maintenance hypotheses, and the older-adult variability they were created to explain, make clear that it is now time to assess their efficacy in explaining the ERP/cognitive-aging data that have been and will be recorded in the future. To conclude, understanding the basis of individual differences and how to exploit them to enable older adults to maintain their EM function at a high level are important goals for future investigations of the neurocognitive aging of memory.

## Conflict of Interest Statement

The authors declare that the research was conducted in the absence of any commercial or financial relationships that could be construed as a potential conflict of interest.
